# Adaptation of Global Standards of Comprehensive Sexuality Education in China: Characteristics, Discussions, and Expectations

**DOI:** 10.3390/children10020409

**Published:** 2023-02-20

**Authors:** Wenli Liu, Jiayang Li, Hongyan Li, Haoran Zheng

**Affiliations:** 1 Collaborative Innovation Center of Assessment for Basic Education Quality, Beijing Normal University, Beijing 100875, China; 2 Independent Researcher, Beijing 100050, China; 3 China Country Office, The United Nations Population Fund, Beijing 100600, China

**Keywords:** China, comprehensive sexuality education, adaptation, guideline, international standards, local context, children, adolescents, youth

## Abstract

This is a project report to introduce the Comprehensive Sexuality Education Technical Guideline—Adaptation of Global Standards for Potential Use in China (First Edition) (hereafter referred to as the Guideline) as the first adaptation of the International Technical Guidance on Sexuality Education (ITGSE) in China. The project was supported by the United Nations Population Fund (UNFPA) and the United Nations Educational, Scientific and Cultural Organization (UNESCO) from 2018 to 2022. The development process included rounds of participatory consultation, validation, and revisions led by the project team and a group of technical advisers from diverse backgrounds. The Guideline is developed to respond to the increasing demands of a technical tool integrating international standards and local context that can be used by all comprehensive sexuality education (CSE) stakeholders in China. While keeping the structure of the ITGSE, the Guideline made modifications and additions in light of the up-to-date Chinese policies, laws, and relevant national programs, as well as Chinese culture and social norms. It is expected that the Guideline could be widely acknowledged, distributed, and used to inform and support the future development of CSE in China.

## 1. Background

According to the definition stated in the International Technical Guidance on Sexuality Education (ITGSE), Comprehensive Sexuality Education (CSE) is a “curriculum-based process of teaching and learning about the cognitive, emotional, physical and social aspects of sexuality [1] (p. 16).” Globally, CSE is recognized as a critical method to achieve universal access to sexual and reproductive health (SRH) and quality education. China, along with other 178 countries in the world, adopted the International Conference on Population and Development (ICPD) Programme of Action in 1994 [2], which commits to promoting adolescents’ well-being through sexuality education. Though great progress has been made in China in terms of CSE implementation in the past decades, many children and adolescents still lack access to quality and affordable CSE that is age-appropriate and scientific, leaving them vulnerable to sexual coercion, sexually transmitted diseases, and unintended pregnancy.

In China, sexuality education is not provided as an independent subject in the school curriculum, but rather, integrated into other relevant subjects of the national school curriculum, such as Physical Education and Health, Biology, Ethics and Rule of Law, and Science. In response to pressing health and social problems, such as unintended pregnancy, different forms of violence, and sexually transmitted infections, including HIV among young Chinese people, however, the delivery of CSE is far from being adequate in China, the primary reason being the absence of national-level technical guidelines and implementation disparity at the community and school levels.

In 2018, the United Nations Educational, Scientific and Cultural Organization (UNESCO) and other UN agencies jointly published the updated edition of the ITGSE [3]. It aims to support education, health, and other relevant authorities in developing and implementing school-based and out-of-school CSE programs and materials. The ITGSE complies with international agreements, instruments, and standards related to CSE with updated evidence, reaffirming “the position of sexuality education within a framework of human rights and gender equality [3].” Since its first publication in 2009, the ITGSE served as an evidence-informed technical resource that is globally applicable and easily adaptable to local contexts with recognition of cultural diversity [3].

In July 2019, the State Council of the People’s Republic of China released the Healthy China Initiative (2019–2030), which established the strategic goals of raising the level of health literacy of the whole population. It outlines the need to strengthen health education, “to integrate health education into the national education system and make health education an important part of quality education at all educational levels. Focus on primary and secondary schools and establish a mechanism to promote health education in schools [4].” Following the long-term national strategy, China made a major commitment to protecting and enhancing young people’s health and well-being by releasing its revised Law on Protection of Minors (revised in 2020) in 2021. The Law states for the first time that “schools and kindergartens should provide age-appropriate sexuality education to minors [5].” The Outline of Children’s Development in China (2021–2030) specified measures and goals to improve the accessibility of sexuality education and sexual health services to school-aged children [6].

The ITGSE was translated into Chinese in 2018 by UNESCO Beijing Office and was jointly launched by the UNESCO Beijing Office and the United Nations Population Fund (UNFPA) China office to introduce global good practices to Chinese readers. However, gaps remain between the generalized international framework with detailed and tailored recommendations for operationalizing CSE in China, where great geographical, cultural, ethnic, social, economic, and political diversity exists. In November 2022, under the support of UNFPA China and UNESCO Beijing Office, the Comprehensive Sexuality Education Technical Guideline –Adaptation of Global Standards for Potential Use in China (First Edition) (hereafter referred to as the Guideline) [7] was released for free public access, after four years of development through a participatory process led by Professor Wenli Liu of Beijing Normal University and a team of national experts.

The Guideline is an adaptation of the ITGSE according to the current evidence on CSE in China, in full consideration of China’s laws, policies, national programs, and social norms on children, young people, and women’s development. It intends to serve as a technical handbook for policymakers, teachers, health educators, program managers, youth development professionals, and young leaders in China in the design, development, implementation, monitoring, and evaluation of CSE curricula and programs. Ultimately, it is committed to ensuring universal access to quality education and health, including sexual and reproductive health information and education for children and young people as they grow [7].

## 2. Development Process and Methods

The project of the Guideline development, supported by UNFPA China and UNESCO Beijing Office, lasted four years from 2018 to 2022. It uses the ITGSE as the main reference and made adaptations according to relevant national policies and guidelines including the School Health Education Guideline, the National Family Education Guideline (revised), and related curriculum standards. The adaption work was undertaken by the project team of Beijing Normal University led by Professor Wenli Liu, backed up by a technical advisory group consisting of researchers and practitioners specializing in sexuality education, laws, public health, psychology, medical education, and sociology. Mixed methods of literature review, stakeholders’ consultations, and validation were used throughout the development process, which can be summarized in three phases: needs analysis, research and development, and release for public access [7].

In the needs analysis phase (2018), the project team conducted an extensive desk review and expert consultation to thoroughly map and analyze relevant research findings and case studies of projects and activities by various organizations as well as issues under heated public discussion. This phase established the need for a technical guideline on CSE relevant to China’s context [7] (pp. 13–19).

The second phase (2019–2021), i.e., the research and development phase, consisted of three steps: (1) review of research literature and national policies. Over one thousand published research papers and articles were reviewed, with more emphasis given to research conducted in China. A comprehensive review of relevant national policies over the decades was also conducted to identify important evidence of political support and guidance; (2) drafting of the framework of the content consisting of key concepts, topics, key points, and learning objectives according to age groups. The project team and the technical advisory group conducted a few rounds of drafting, discussion, and revision of the content framework, aiming to fully reflect the key characteristics of successful sexuality education programs—scientific accuracy, logic, continuation, consistency, comprehensiveness, and age-appropriateness [7] (pp. 18–19); (3) multi-stakeholder consultation. A series of consultation meetings both online and offline were conducted to get further inputs and feedback on the draft Guideline. A multi-stakeholder CSE stakeholder workshop was organized in Beijing in 2019 by the project team with support from UNFPA and UNESCO, involving over one hundred participants who provided valuable feedback for further improvement of the Guideline [8].

In the third phase in 2022, the first edition of the Guideline was finally completed and endorsed by UNFPA and UNESCO after incorporating the stakeholders’ final comments. The Guideline, originally developed in Chinese, has also been translated into English. Both versions are released by UNFPA on its official website for open and free access [9]. UNFPA and UNESCO expect to work with a Chinese institutional partner to launch and distribute the Guideline as a technical resource among policymakers, teachers, health educators, program managers, youth development professionals, and young leaders in China to inform the design, implementation, and monitoring and evaluation of quality CSE curricula and programs.

Something worth noting is that, during the development process of the Guideline, significant policy changes took place with a positive impact on sexuality education in China. Right after the launch of the Chinese translation of the ITGSE in 2018, the National Health Commission issued the Key Messages and Interpretation of Adolescent Health Education in China. It put forward nine key messages, one of which states that it is necessary to “master correct reproductive and sexual health knowledge, avoid premature sexual behavior, and prevent sexually transmitted diseases such as AIDS [10].” The interpretation of this key message directly points to “comprehensive sexuality education” as an important measure to improve teenagers’ cognition of SRH, responsibility for self-protection, and respect for others’ physical and mental health. That represents the first official use of the term CSE in China. However, the most significant change came in 2020 when the revision of the national Law on the Protection of Minors legalized ‘sexuality education’ for the first time in Chinese history [5]. Following that, both the Outline of Children’s Development in China (2021–2030) [6] launched by the State Council and the Regulation on School Protection of Minors by the Ministry of Education in 2021 [11] included ‘sexuality education’ as an action. During the public and/or expert consultation phases of these important laws and policies, the technical advisory group and project team for the Guideline development played an active role in advocating for the inclusion of sexuality education. In a way, the development process of the Guideline and the policy change trajectories went side by side, and at a certain point, they came across and benefited each other.

## 3. Results and Characteristics

In terms of the content framework, the Guideline very much resembles the ITGSE, maintaining the structure of eight key concepts with three-dimensional learning objectives (knowledge, attitudes, and skills) and four age groups. A slight adjustment was made to age groups, i.e., the age group of 5–8 in ITGSE was changed to lower primary (6–9), and the other three age groups remain the same but were renamed as upper primary (9–12), lower secondary (12–15) and upper secondary (15–18 and above), in correspondence with the Chinese schooling system. Like the ITGSE, the Guideline also follows a positive approach to sexuality, and fully reflects the principles of rights and gender equality.

It is important to note that the adaptation was made based on the Chinese language edition of the ITGSE, and some minor changes are no more than calibration of vocabulary. Compared to the ITGSE, the Guideline demonstrates a certain level of adjustments and modifications as a result of the adaptation process. The eight key concepts remain the same, with only Key Concept 2 changed from “Values, Rights, Culture and Sexuality” to “Values, Rights, Culture, Media and Sexuality” (refer to Table 1). Other major changes are summarized below, highlighting the differences related to the learning objectives and key ideas [7].

### 3.1. Added Content Related to the Legislation

Content related to the legislation was added and fully reflected in the framework in terms of the topics, key ideas, and learning objectives (refer to Table 1 and Table 2). Family planning has been made a basic national policy since 1982 [12]. In the Guideline, relevant content on legislation is added to help learners realize the importance of SRH and be aware of their rights in relation to SRH and relevant services as embodied in the National Population and Family Planning Law [13] launched in 2001. More emphasis is given accordingly to guide the learners to understand the impact of laws on individual sexual behavior and acquire help-seeking skills, especially the laws and regulations regarding the prevention of bullying and violence, child abuse, sexual abuse, and intimate partner violence. For example, this emphasis is made by adding “may even be illegal and criminal” after every key idea of topic 4.1—Violence, for the lower primary stage [7] (p. 57). Considering the implication of fast-developing digital technologies on the lives of young people, a learning objective regarding the legal regulations on the use of the Internet and social media was added to Topic 4.4—Safe Use of Information and Communication Technologies (ICTs) [7] (p. 62) (refer to Table 2). The latest statistics [14] show that the prevalence rate of Internet use among minors has reached 94.9%, which is higher than the national average of 70.4%, and the age of the digital population is continuously decreasing. Government regulation on the cyber protection of minors is under development right now [15] in an effort to support the implementation of the national Law on Protection of Minors. Another added objective for lower primary students regarding male responsibility in the pregnancy process highlights the importance for learners to equip a gender-equitable attitude in parenting (refer to Table 2). Men’s parenting responsibility is an area that has been increasingly receiving policy and social attention, in terms of men’s entitlement to parental leave and men’s role in aristogenesis fine rearing, for example, by reducing smoking in the presence of pregnant women.

### 3.2. Added Content in Response to Social Topics under Hot Discussion

In recent years, a number of sexuality-related topics have attracted public attention, e.g., human trafficking, sexual abuse, bullying, sexual consent, stigma, and myths regarding menstruation and nocturnal emissions. More key ideas and learning objectives about these were added to the Guideline (refer to Table 2).

The phenomenon of human trafficking dies hard. In early 2022, the news about a trafficked woman giving birth to eight children in a village in Fengxian of Jiangsu province aroused wide social attention [16]. Human trafficking is strictly prohibited by Chinese law, and major actions are being taken by the government [17]. The newly adopted revision of the Law on Protection of Women and Children of the People’s Republic of China [18] puts more emphasis on the protection of women’s rights to life, body, and health, as well as the prohibition of women trafficking, stressing the accountability of all relevant government departments and organizations and individual citizen’s responsibility in non-discrimination of trafficking victims. A key idea of “criminal behavior of women trafficking shall be resolutely prohibited” was thus added under topic 1.4—Long-Term Commitment and Parenting [7] (p. 35), to help learners acquire the necessary knowledge, attitudes, and skills in understanding the phenomenon of women trafficking and develop an awareness of conscious actions (refer to Table 2).

Child sexual abuse (CSA) has been a heated debate topic in recent years, with more cases being exposed by the media and relevant statistics widely publicized by active non-governmental organizations (NGOs). Sexuality education was legalized by the Law on Protection of Minors to help reduce CSA. However, CSA is a controversial area, and it is closely related to sexual consent, which the latter became another heatedly discussed social topic when the alleged case of a male lawyer abusing a teenage girl was exposed in 2020 [19,20]. There is no clear definition of the age of sexual consent in Chinese laws, but the Criminal Law (revised in 2020) stipulates that “adultery with a young girl under the age of fourteen shall be regarded as rape and receive heavier punishment” (Article 236) [21]. In response to this, the Guideline made ‘consent’ a separate topic by breaking down the original topic 4.2-Consent, Privacy and Bodily Integrity into two topics, i.e., 4.2-Consent and 4.3-Privacy and Bodily Integrity, to allow the addition of more specific learning objectives [7] (pp. 59–61) (refer to Table 1).

School violence bullying is another issue of major concern in recent years. In 2016 the State Council ordered a special inspection on school bullying, and in 2017, eleven government departments jointly issued a comprehensive plan of control [22]. Therefore, the Guideline added one key idea about school bullying under topic 4.1-Violence [7] (p. 57) (refer to Table 2).

Another area further strengthened in the Guideline is stigma and misunderstanding about menstruation and nocturnal emissions. In early 2020 during the initial stage of the COVID epidemic, a China Central Television (CCTV) interview with a female nurse in Wuhan purposely cut a 31-s segment of the nurse saying, “I was suffering from abdominal pain due to menstruation,” causing hot discussion in the public about the menstrual stigma [23]. Another news [24] in 2022 about a female passenger unable to buy sanitary pads on the train inspired another round of public debate, resurfacing the widespread social and cultural attitudes about sexuality. Similarly, there are many myths about nocturnal emissions, with some unfounded traditional beliefs about their negative impact on fertility, longevity, and physical health. A specific learning objective was added to the Guideline to reflect this obvious social need [7] (refer to Table 2).

### 3.3. Strengthened Content about Gender Equality

The concept of sexual identity and sexual expression are hardly seen in primary education in China. Recognizing the importance of understanding these concepts for girls and boys entering puberty, the Guideline added quite a few key ideas regarding sexual diversity, to reduce bias, stigma, and discrimination against the sexual minority population (refer to Table 2). Sexual minority students, especially transgender students, are at higher risk of various forms of school bullying due to their sexual orientation and identity, which negatively impact their academic achievements, physical and mental health, and well-being [25]. Studies [26] show that students learning about homosexuality in primary school are more tolerant of sexual minorities than students learning about it only in lower or upper secondary schools, highlighting the importance of providing such education at an early stage. Learning about sexual orientation before puberty starts also saves students from the plethora of erroneous information in society and prevents them from unfounded fear about self-identity of homosexuality. Evidence-based research shows that providing CSE at an early age helps reduce gender stereotypes for children as they grow up and is critical in eliminating gender-based violence (GBV) [27,28,29]. GBV was further highlighted in the Guideline by the addition of a key idea for upper secondary students to build their understanding of the social construction of gender norms, relevant laws and policies, and response strategies (refer to Table 2).

### 3.4. Responding to Customary Practices in the Chinese Culture

In responding to cultural and customary practices regarding social relationships, the Guideline added ‘mercenary marriage’ (refer to Table 1) to the key idea regarding child, early, and forced marriages under the topic 1.4-Longterm Commitment and Parenting [7] (pp. 35–36). Dowry is still in practice in many places in China, which commonly far exceeds the local average cost of living. Although dowry is not legally banned, it is a muddled area that borders on extorting in the name of marriage.

It is necessary for students to be aware of the existence of early, forced, and mercenary marriage, which is against the principle of freedom of marriage, and to be able to seek support when necessary. Early marriages and parenting still exist and affect adolescents’ lives, putting their SRH at risk. Statistics [30] show that 12.2% of males and 13.7% of females marry before the legal marriage age. In 2015, the fertility rate of female adolescents, predominantly 18 and 19-year-olds, was 9 per thousand. The fertility rate of 19-year-old rural female adolescents is as high as 37%. In remote ethnic minority areas, affected by both local traditions and environment, the practice of early marriage and parenting as well as male preference still prevails. Therefore, students must understand the root cause of early childbearing and be able to show empathy to adolescent girls experiencing early marriage and parenting.

### 3.5. Further Stressing of the Positivity of Sexuality

It is natural for adolescents to have sexual fantasies and sexual dreams [31], but the lack of scientific understanding of this phenomenon causes negative reactions of shame, self-blame, fear, and pessimism among adolescents. There is also misunderstanding and confusion about masturbation. Studies [31,32,33] show that masturbation is common among adolescents, but a significant proportion of adolescents regard masturbation as a bad habit, disease, or criminal behavior and suffer from mental distress because of that. Therefore, two key ideas were added to enable students to have the right understanding of sexual fantasies and masturbation [7] (refer to Table 2).

### 3.6. Information about Resources and Support from Both the Government and Civil Society

Prevention and control of HIV/AIDS have been high on the Chinese government’s agendas. The Healthy China 2030 Initiative (2019-203) [34] puts forward the strategy of prioritizing prevention for major diseases such as AIDS. It includes the objective of keeping HIV/AIDS at a low epidemic level. The Regulation on HIV Prevention and Control (revised in 2019) [35] stipulates the national policy of free HIV counseling and testing. Many people are not aware of these laws, regulations, and policies regarding HIV/AIDS prevention and control, hence the addition of two key ideas under the topic 8.4-HIV and AIDS-Stigma, Treatment and Support [7] (p. 97) (refer to Table 2). The human papillomavirus (HPV) vaccine was introduced in China several years ago, but the supply remains inadequate, and the vaccination rate is low. With more areas starting to promote free and subsidized HPV vaccination, including among middle school students, it is necessary to raise awareness about HPV vaccination through sexuality education and encourage participation. A specific learning objective regarding HPV vaccination is therefore added under the topic of STI [7] (p. 95) (refer to Table 2).

## 4. Discussions and Expectations

The Guideline is an adaptation of the ITGSE in China, with adequate consideration of the latest situation, social discussion, cultural traditions, national policies, and available support services. The adaptation was conducted by sexuality education experts and technical advisors in China with diver expertise and experiences through a long and iterative process including desk research and extensive multi-sectoral consultation.

In the process of developing the Guideline, the relevant policies on sexuality education in China have undergone significant changes. The forces jointly mobilized by the project team, the technical advisory group, and the UN agencies during the compiling, consulting, drafting, and validating process contributed to policy advocacy. At the same time, these policies have guided the development of the Guideline. Some of the technical advisory group members also played an influential role in influencing policies by providing strong technical inputs. With the legalization of sexuality education through the revised Law on Protection of Minors and the mandating of sexuality education by the Ministry of Education in its new policy on school protection, sexuality education has been moved one level higher on the government’s agendas. Though both documents positioned sexuality education as a strategy to prevent sexual abuse and harassment, the exact naming of ‘sexuality education’ was significant enough to raise the level of acceptance and inspire public discussion about the implementation of sexuality education in its broader sense.

When it comes to the actual implementation of sexuality education in schools, the most relevant education policy is the “Guide of Integrating Life Safety and Health Education into Primary and Secondary School Curriculum Textbooks [36]” issued by the Ministry of Education in 2021. It broadened “growth and development and adolescent health care” (one of the five content areas identified in the Health Education Guidelines for Primary and Secondary Schools) into five aspects: growth and development, adolescent psychology, adolescent sexual health, sexual assault prevention, and cherishing life. It guides how these contents could be integrated into and delivered through the current national school curriculum. Although it did not use the term “sexuality education”, it is by far the most direct and practical guide from the national level regarding the actual implementation of sexuality education in schools.

The Guideline, as a collective brainchild of national experts, is undoubtedly a driving force to inform and support the work of sexuality education practitioners in China, including curriculum developers and educators in schools and out-of-school settings. However, how likely is the Guideline to be adopted by the Ministry of Education of China? Or would the Guideline be acknowledged as a practical and relevant tool to inform the development of future educational policies? With the absence of direct support and endorsement from the national education authority in the very beginning, a long journey ahead shall be expected for an official guideline of a similar nature, but hopefully, it will be a journey made shorter compared to one with the absence of such an adaptation process.

It was expected by UNFPA and UNESCO that the Guideline could be jointly launched by a national-level institution in China to showcase a formal acceptance. In 2022, no such institutions were identified or confirmed for a joint launch. The Guideline was then released online for the public’s free access in November 2022, followed by a Train of Trainers (TOT) workshop led by the project team and supported by UNFPA China [37] in December 2022. A total of 30 CSE practitioners, including program managers and local schoolteachers from different areas of China [38], were selected to join the four-day participatory TOT. They were the first group of CSE trainers to systematically study the Guideline and are expected to align their work to benefit more young people in a standardized and holistic way. Furthermore, to provide concrete evidence and support for the Guideline to be recognized by law enforcement departments or for wider adoption, it is also recommended that CSE practitioners could conduct follow-up actions in the future, including but not limited to generating qualitative and quantitative data through research, pilots, advocacies, and programs.

## Figures and Tables

**Table 1:** Modifications Made to the ITGSE.

Serial No.		ITGSE	The Guideline	Remarks
1	Key concept	2. Values, Rights, Culture and Sexuality	2. Values, Rights, Culture, Media and Sexuality	
2	Topic	2.3 Culture, Society and Sexuality	2.3 Culture, Society, Law and Sexuality	
3		4.2 Consent, Privacy and Bodily Integrity	4.2 Consent; 4.3 Privacy and Bodily Integrity	
4	Key idea	It is important to be aware of how social and cultural norms impact sexual behavior while developing one’s own point of view (upper secondary).	Further learn about and reflect on how social, cultural, and legal norms impact sexual behavior, and form and develop one’s own perspectives based on this (upper secondary)	Topic 2.3 Culture, Society, Law and Sexuality
5	Key idea	All forms of GBV by adults, young people, and people in positions of authority are a violation of human rights.	All forms of GBV by adults, young people, and people with special responsibilities for minors are a violation of human rights.	Topic 3.3-Gender-based Violence
6	Key idea	Child, early, and forced marriages (CEFM) are harmful and illegal in the majority of countries	Child, early, forced and mercenary marriages are harmful and illegal in the majority of countries including China	Topic 1.4- Long-term Commitment and Parenting

**Table 2:** Newly Added Contents.

Newly Added Key Ideas		
1	Children enjoy various rights (lower primary);	Topic 2.2 Rights and Sexuality
2	Human rights outlined in China’s national laws and international agreements (upper primary);	As above
3	Laws influence people’s perceptions and attitudes about sexuality-related issues (lower secondary);	Topic 2.3—Culture, Society, Law and Sexuality
4	Resolutely resist the crime of trafficking in women (lower secondary)	Topic 4.1 Violence
5	Resolutely resist the crime of child trafficking (lower secondary)	As above
6	Identify child sexual abuse and acknowledge that this is wrong and may even be illegal and criminal (lower primary)	As above
7	School bullying of any kind is not to be tolerated (upper primary)	As above
8	Learn about gender identity and gender expression (lower primary)	Topic 3.1—The Social Construction of Gender and Gender Norms
9	Human sexual orientations are diverse (upper primary)	As above
10	Understand sexual minorities (upper primary)	As above
11	Understand the diversity of sexual orientation (lower secondary)	As above
12	Homophobia and transphobia are harmful to people of diverse sexual orientations and gender identities (lower secondary)	As above
13	Gender has a profound impact on the establishment and functioning of social systems (upper secondary)	As above
14	LGBTI people can decide their way of life under the precondition of not harming themselves and others (upper secondary)	As above
15	Respect LGBTI people (upper secondary)	As above
16	Gender inequality, social norms, and power differences affect sexual intercourse and may increase the risk of sexual coercion, abuse, and other forms of GBV (upper secondary)	and Topic 3.3—Gender-based Violence
17	Sexual fantasies and dreams during puberty are natural (upper primary)	Topic 7.2 Sexual Behavior and Sexual Response
18	Masturbation is natural and does not cause harm to the body (upper primary)	As above
19	Centers for AIDS Prevention and Control and local Centers for Disease Control and Prevention can provide free HIV and AIDS counseling and testing services (lower secondary)	Topic 8.4 HIV/AIDS Stigma, Treatment, Care and Support
20	China’s policies on AIDS prevention and control will help people attach more importance to and effectively participate in AIDS prevention and control actions (upper secondary)	As above
**Newly Added Learning Objectives**		
1	Be able to advocate for national laws and regulations that support sexual and reproductive health rights (skills, upper secondary)	Topic 2.2—Rights and Sexuality
2	Able to advocate for the promotion and protection of human rights in family planning policies and related services (skills, upper secondary)	Topic 2.3—Culture, Society, Law and Sexuality
3	Learn about China’s laws and regulations on the ruse of the Internet and social media by minors (knowledge, upper primary)	Topic 4.4—Safe Use of ICTs
4	Acknowledge that men should also take responsibility in the process of conceiving a new life (attitude, lower primary)	Topic 6.2—Reproduction
5	Be able to reflect on the stigma of menstruation and misconception about ejaculation (including seminal emission) that arise in the family, school, community, and society, and able to help others develop scientific perceptions (skills, upper primary)	Topic 6.1—Sexuality, Reproductive Anatomy and Physiology
6	Show empathy to people who give birth too early (attitude, lower secondary)	Topic 8.1—Pregnancy
7	Be able to reflect on the cause of too early child-bearing (skills, lower secondary)	As above
8	Describe at what age and where the vaccine for genital human papillomavirus (HPV) can be administered, if such a vaccine for the prevention of cervical cancer and other diseases is available locally (knowledge, upper primary)	Topic 8.3—Sexually Transmitted Infections (STIs), including HIV

## Data Availability

Not applicable.

## References

[1] UNESCO (2018). International Technical Guidance on Sexuality Education.

[2] UNFPA (1994). Report of the International Conference on Population and Development.

[3] UNESCO UN Urges Comprehensive Approach to Sexuality Education. https://en.unesco.org/news/urges-comprehensive-approach-sexuality-education.

[4] Central Committee of Chinese Communist Party, State Council (2016). The Plan for Healthy China 2030.

[5] The National People’s Congress of the People’s Republic of China (2020). Law of the People’s Republic of China on Protection of Minors.

[6] Xinhua News. http://english.www.gov.cn/policies/latestreleases/202109/27/content_WS61516ea9c6d0df57f98e0f0f.html.

[7] UNFPA, UNESCO (2022). Comprehensive Sexuality Education Technical Guideline—Adaptation of Global Standards for Potential Use in China.

[8] UNFPA China National Consultative Meeting Held to Improve China’s Comprehensive Sexuality Education Guidance. https://china.unfpa.org/en/news/190822.

[9] UNFPA China Comprehensive Sexuality Education Technical Guideline: Adaptation of Global Standards for Potential Use in China (First Edition). https://china.unfpa.org/en/publications/22110701.

[10] National Health Commission of the People’s Republic of China The Second Distribution Material of the National Health Commission’s Regular Press Conference on 25 September 2018: Core Information and Interpretation of Chinese Youth Health Education (2018 Edition). http://www.nhc.gov.cn/wjw/zccl/201809/820dd3db393c43c1a230817e2e4b9fd5.shtml.

[11] Ministry of Education of the People’s Republic of China The Regulation on School Protection of Minors. http://www.moe.gov.cn/srcsite/A02/s5911/moe_621/202106/t20210601_534640.html.

[12] Women Global Gender Equality Constitutional Database Constitution of the People’s Republic of China 1982, as Amended to 2018. https://constitutions.unwomen.org/en/countries/asia/china?provisioncategory=b023d3e9cea2418facb51f5115028c03.

[13] The National People’s Congress of the People’s Republic of China (2021). The Population and Family Planning Law of the People’s Republic of China.

[14] The Communist Youth League. https://pic.cyol.com/img/20210720/img_960114c132531c521023e29b6c223e438461.pdf.

[15] Cyberspace Administration of China Cyberspace Administration of China’s Second Notice for Public Comments on the Regulation on Cyber Protection of Minors (Draft for Comments). http://www.cac.gov.cn/2022-03/14/c_1648865100662480.htm.

[16] Bureau of Information of the Supreme People’s Court Investigation into the Incident of “A Woman Who Gave Birth to Eight Children in Fengxian County. https://baijiahao.baidu.com/s?id=1725573554886874922&wfr=spider&for=pc.

[17] The State Council of the People’s Republic of China Notice by the General Office of the State Council of Issuing China’s Action Plan against Human Abduction and Trafficking (2021–2030). http://www.gov.cn/zhengce/content/2021-04/28/content_5603574.htm.

[18] The National People’s Congress of the People’s Republic of China (2022). Law on Protection of Women and Children of the People’s Republic of China.

[19] The Paper Bao Yuming was Suspected of Sexually Assaulting His “Adopted Daughter,” the Police Notified Again in the Early Morning: A Special Working Force has been Established. https://m.thepaper.cn/baijiahao_6911535.

[20] The Paper Latest|The Supreme Procuratorate and the Ministry of Public Security Intervene in the “Senior Executive Sexual Assault on Adopted Daughter Case”. https://m.thepaper.cn/baijiahao_6954753.

[21] The National People’s Congress of the People’s Republic of China (1997). Criminal Law of the People’s Republic of China.

[22] The State Council of the People’s Republic of China Eleven departments including the Ministry of Education Jointly Issued the “Strengthening the Comprehensive Governance Plan for Primary and Secondary School Students Bullying”. http://www.gov.cn/xinwen/2017-12/28/content_5251115.htm.

[23] Qin S., Liu T., Jiang Y., Yang T., Huang L., Jiang N., Lv J. Central China Normal University. http://media-ethic.ccnu.edu.cn/info/1189/2447.htm.

[24] Tencent News A Woman Whose Period Suddenly Came Couldn’t Buy Sanitary Napkins. Railway: Personal Items are Not Sold, Sparking Heated Discussions Online. https://view.inews.qq.com/wxn/20220917A05U2L00?qq=1839625204.

[25] Wei C., Liu W. (2015). A Study on the Relationship Between the Mental Health and School Bullying Experience of Sexual Minority Students. Chin. J. Clin. Psychol..

[26] Guo L., Liu W. (2019). Education about Diversity of Sexual Orientations in Comprehensive Sexuality Education. Jiangsu Educ..

[27] Li J., Liu W. (2020). Gender Stereotypes and Gender Equality Education. Jiangsu Educ..

[28] Liu J., Liu W. (2019). Evaluation of the Effectiveness of Primary School Sexuality Education based on Sexuality Knowledge and Gender Stereotypes. Chin. J. Sch. Health.

[29] Bian L., Leslie S.J., Cimpian A. (2017). Gender Stereotypes about Intellectual Ability Emerge Early and Influence Children’s Interests. Science.

[30] Peking University Open Research Data China Family Panel Studies. https://opendata.pku.edu.cn/dataset.xhtml?persistentId=doi:10.18170/DVN/45LCSO.

[31] Jing X., Zheng Y. (2008). A Study on Sexual Dreams and Sexual Fantasy of Students of a Vocational School. Med. Soc..

[32] Cui G., Bai W., Gao Z., Li Q., Sun H. (1997). Status and Analysis of Sexual Dreams During Puberty. J. Hebei Norm. Univ..

[33] Xu M., Shao P. (2005). Analysis of Sexual Fantasy and Sexual Dream among 908 Students of Ningxia University. Chin. J. Sch. Health.

[34] The Health China Initiative. https://www.jkzgxd.cn/.

[35] (2019). The State Council of the People’s Republic of China: AIDS Prevention and Control Regulations.

[36] Ministry of Education of the People’s Republic of China (2021). Guide of Integrating Life Safety and Health Education into Primary and Secondary School Curriculum Textbooks.

[37] UNFPA Call for Applications: National Individual Consultant for conducting an online TOT workshop using the CSE Technical Guideline—Adaptation of Global Standards for Potential Use in China (First Edition). https://china.unfpa.org/en/vacancies/22101302.

[38] Love and Life Work Brief of Liu Wenli Sexuality Education Working Group in December 2022. https://mp.weixin.qq.com/s/TyZVZ4hO7hUNVpZHKVHw4w.

